# Supplementary material for: Genomic epidemiology of Streptococcus pyogenes from pharyngeal and skin swabs in Gabon
Source: Microbiol Spectr. 2024 May 24;12(7):e04265-23. doi: 10.1128/spectrum.04265-23 (PMC11218484; doi:10.1128/spectrum.04265-23)

**Supplementary Figure S1. Comparison of the clustering between SNP-based (≤18SNPs) and Average nucleotide identity based (≥99.99%) clustering.**

**
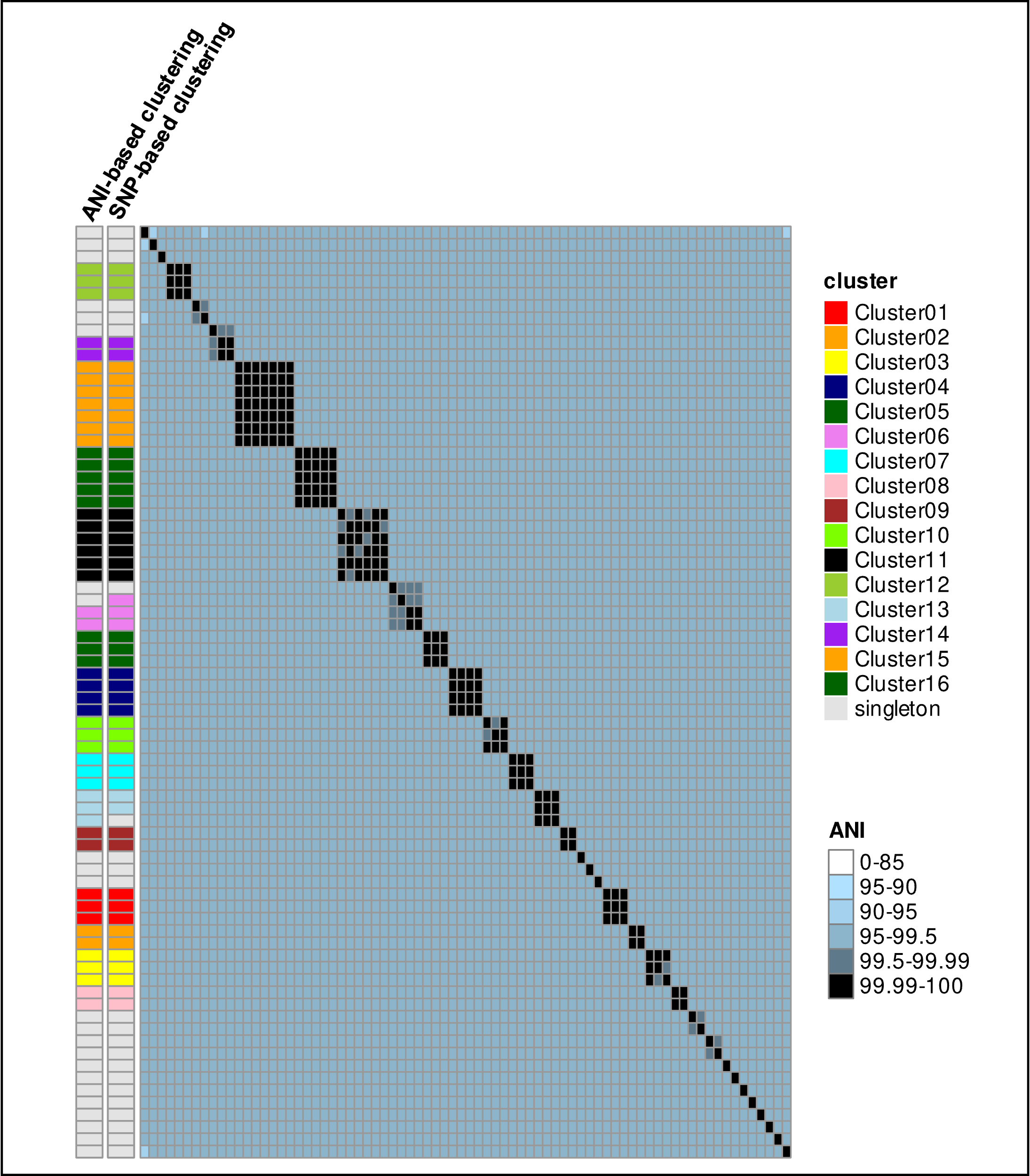
**

**Supplementary Figure S2. Comparison of *emm*-typing results by microarray and whole-genome sequencing.**

**
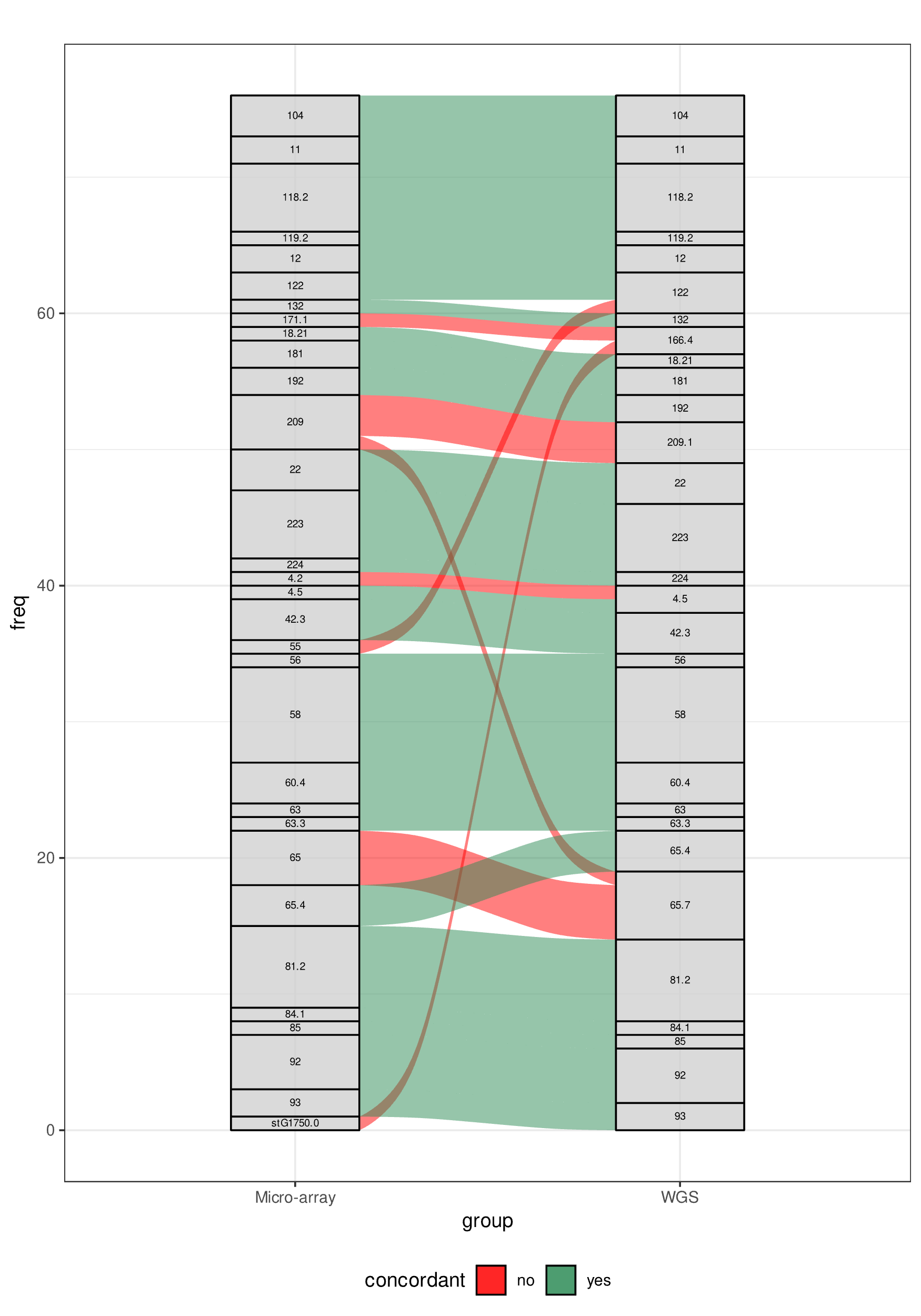
**

**Supplementary Figure S3. Virulome of the *S. pyogenes* isolates from Gabon**


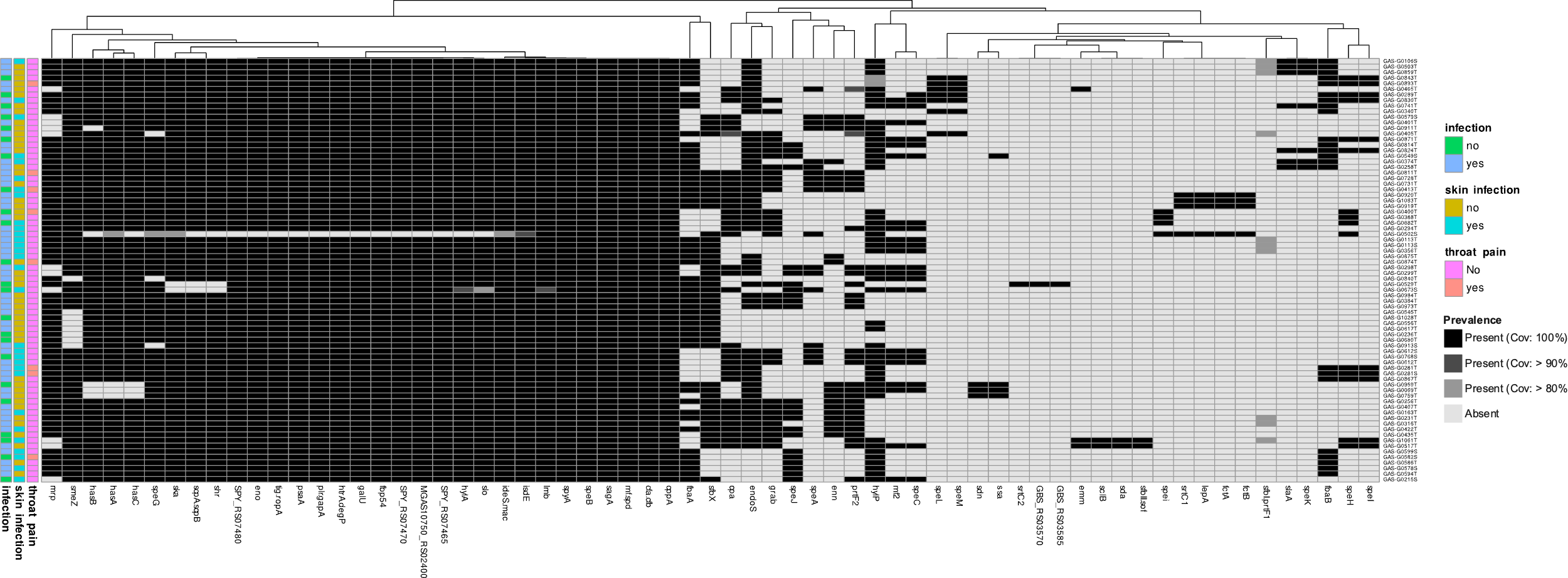


**Supplementary Figure S4: Prevalence of *S. pyogenes* *emm*-types worldwide**


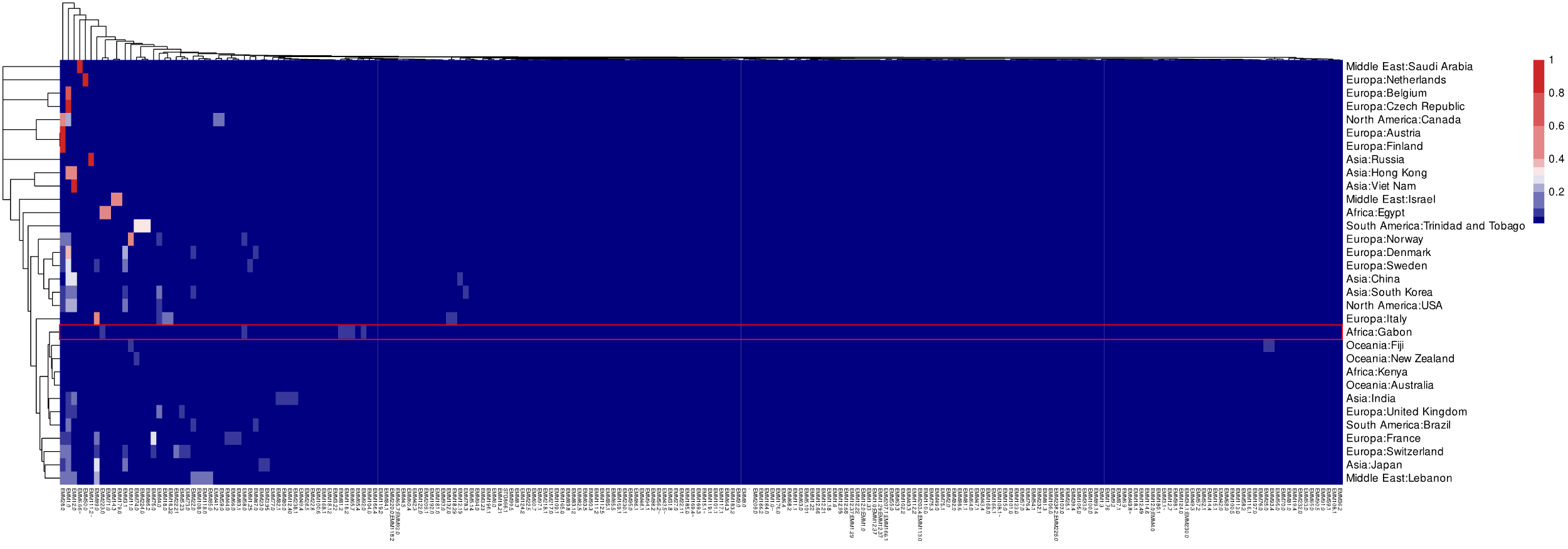


**Supplementary Figure S5: Prevalence of *S. pyogenes* e*mm*-clusters worldwide**


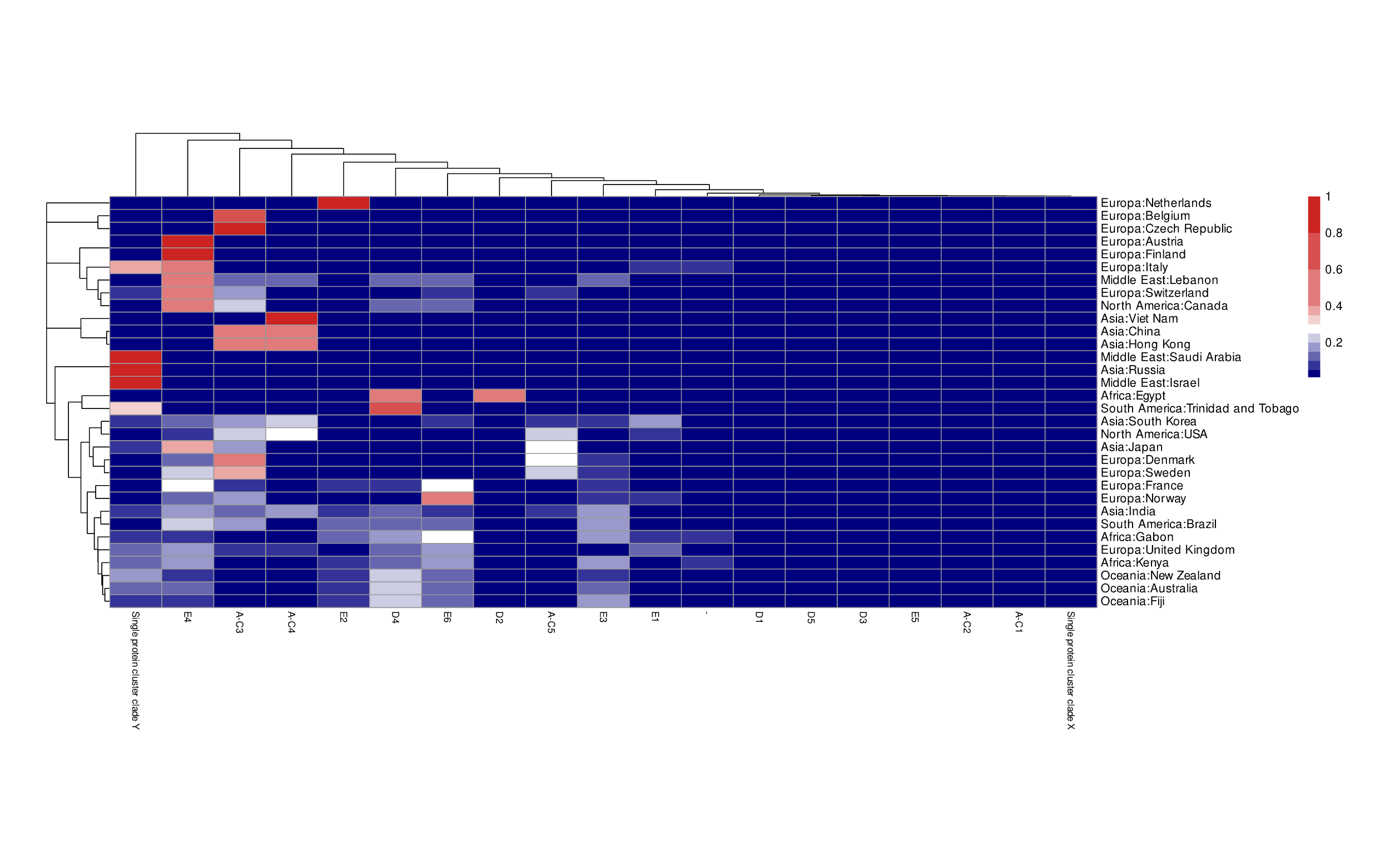

Supplement: Supplemental material — Fig. S1 to S5. [file spectrum.04265-23-s0001.docx]
